# Molecular and serological survey of paratuberculosis in cattle in selected districts of Western Uganda

**DOI:** 10.1186/s12917-022-03535-7

**Published:** 2022-12-14

**Authors:** Judah Ssekitoleko, Lonzy Ojok, Saint Kizito Omala, Mohammed Elwasila Mukhtar, Kamal H. Eltom, El Sagad Eltayeb, Clovice Kankya, Magid Kisekka, Uwe Truyen, Claus-Peter Czerny, Ahmed Abd El Wahed, Julius Boniface Okuni

**Affiliations:** 1 College of Veterinary Medicine, Animal Resources and Biosecurity, Makerere University, Kampala, P. O. Box 7062, Uganda; 2 Department of Livestock Health Research, Rwebitaba Zonal Agricultural Research and Development Institute, National Agricultural Research Organisation, Entebbe, P. O. Box 295, Uganda; 3 Department of Pathology, Faculty of Medicine, Gulu University, Gulu, P.O. Box 166, Uganda; 4 Department of Statistical Methods and Actuarial Science, School of Statistics and Planning, Makerere University, Kampala, P.O. Box 7062, Uganda; 5 Department of Agricultural Extension and Rural Development, Faculty of Agriculture, University of Khartoum, Khartoum, Sudan; 6 Unit of Animal Health and Safety of Animal Products, Institute for Studies and Promotion of Animal Exports, University of Khartoum, Shambat, 13314 Khartoum North, Sudan; 7 Faculty of Medicine, Al Neelain University/Ibn Sina Specialised Hospital, 11112 Khartoum, Sudan; 8 Institute of Animal Hygiene and Veterinary Public Health, Leipzig University, D-04103 Leipzig, Germany; 9 Division of Microbiology and Animal Hygiene, University of Goettingen, D-37077 Goettingen, Germany

**Keywords:** Paratuberculosis, Prevalence, Western Uganda, Recombinase polymerase amplification

## Abstract

Knowledge of *Mycobacterium avium* subsp. *paratuberculosis* (MAP) herd infection status is important to plan appropriate control and prevention strategies for Paratuberculosis (PTB); however, in Uganda MAP infection status of most herds is unknown. This study aimed at determining the MAP infection status of cattle herds and the associated risk factors for MAP infection in six western districts of Uganda. The survey covered a total of 93 herds where faecal and blood samples were collected from 1814 cattle. A Recombinase Polymerase Amplification (RPA) and an antibody-based (ELISA) assays were used to test for the presence of MAP DNA in faeces and MAP antibodies in serum, respectively. The apparent cow-level prevalence of MAP infection was 3.2 and 2.7% using ELISA and RPA respectively and the true cow-level prevalence using ELISA and RPA was 4.9 and 3% respectively. A herd-level prevalence of 43% (ELISA) and 40.8% (RPA) and a within-herd prevalence of 3.8 ± 2.1% based on ELISA were obtained. Among the risk factors investigated, long dry spells were significantly associated with high MAP infection (*p < 0.05*). These results indicate that MAP is actively present in most areas where surveillance was carried out. This poses a serious threat to the livestock industry and potentially to public health as MAP is highly suspected to play a role in the pathogenesis of several diseases in humans. Other areas of the country are to be surveyed as well in order to establish full data on MAP infection status to enable interventions for the control and prevention of the disease.

**Supplementary Information:**

The online version contains supplementary material available at 10.1186/s12917-022-03535-7.

## Background

Paratuberculosis (PTB) or Johne’s Disease (JD) is a disease of significant economic impact on livestock farming worldwide and affects farmed and wild ruminants such as cattle, sheep, goats, buffaloes, bison, alpacas, and deer [1]. The disease has also been reported in other animals like stoats, badgers, camelids, and non-human primates [2, 3]. It has also been reported in a dog [4]. The causative agent, *Mycobacterium avium* subsp*. paratuberculosis* (MAP), has been isolated severally from humans with Crohn’s disease (CD) and ulcerative colitis as well as from many other diseases making it a potential zoonosis [5–7].

The economic cost of PTB is due to deaths, reduced productivity, costs of treatments, reduced growth rate, and lack of market for replacement heifers; especially in countries where Johne’s disease-free state is required [8–10]. Dairy production is one of the sectors grossly affected by paratuberculosis [11, 12].

Control of PTB is very difficult because there are no effective treatments, no vaccine that prevents the infection, and lack of diagnostic methods that offer early detection; and measures to reduce infections are very expensive. Most diagnostic methods in use detect the infections only in animals with advanced clinical disease, when the animal is already spreading the mycobacteria and when no treatments can be effective [13–15] and therefore, are of limited value in control and prevention. Nonetheless, diagnostic tests, play a key role in establishing an effective control program. Knowledge of the prevalence and risk factors of the disease in any locality is key to planning control measures that combine different approaches. The occurrence of PTB has been associated with certain risk factors though these may vary from one geographical region to another, hence the need for surveys in different regions. According to Doré and others [16], contact with faeces from the infected dam was the most important risk factor for MAP transmission to calves; other factors such as calf feeding and housing were all related to contamination with dam faeces. In a study from Ireland, the odds of a positive MAP test were increased with reduced attendance at time of calving [17]. A survey of possible risk factors for MAP in Canadian herds revealed that having more than one cow in the maternity pen, group housing of calves, recent purchase of heifers and herds with comorbidities, such as bovine viral diarrhoea virus were significantly associated with MAP occurrence [18]. In another study from Brazil, [19], failure to use maternity pens was the most significant risk factor associated to MAP transmission.

While knowledge about the prevalence and epidemiology of paratuberculosis and its causative agent are well documented in the developed countries, in Africa, there are still large knowledge gaps around PTB [20].

In Uganda, the prevalence of PTB has been reported in selected districts of the central region [21, 22]. These studies have shown that MAP infection is established in the districts studied but hitherto, there has been no data from other parts of the country, especially in the major livestock producing regions, such as western Uganda, the major supplier of Ugandan’s milk and beef products. Additionally, nothing is known about the epidemiological factors that are associated with MAP infections and transmission in those districts.

The aforementioned studies relied on serology only. Use of both molecular and serological tests in the current study aimed at improving the ability of the diagnostic method to detect MAP in either faeces or blood of the sampled cattle.

This paper describes the prevalence of MAP infection/PTB in selected districts of western Uganda and the risk factors associated to MAP infection. A combination of serologic and molecular methods was used for more accurate estimates of paratuberculosis prevalence.

## Results

A total of 1814 heads of cattle comprising 93 herds from six districts were tested for MAP infection using ELISA and RPA. The cattle population comprised mixed cattle breeds common in Uganda and these included; Friesians and their crosses which were the majority in the survey, Ankole long-horned and Zebu (Table 1).

**Table 1:** Breeds of cattle sampled and tested for paratuberculosis in the six districts of western Uganda

Breed	Number of cattle tested	Number of cattle tested (%)	Number positive with ELISA	Number positive with RPA
Friesians	838	46	28	23
Friesian crosses	534	29.4	18	14
Ankole long-horned	213	12.6	6	8
Zebu	229	12	6	4
**Total**	**1814**		**58**	**49**

Of the 1814 heads of cattle, 58 were ELISA positive giving an apparent cow-level prevalence of 3.2% and 40 of the 93 herds had at least one MAP-positive animal resulting in a herd-level prevalence of 43% (Table 2). The average district seroprevalence was 3.36(±1.25%), being lowest in Kiruhura with 2.0% and highest in Ntoroko with 5.0% and a with-in herd prevalence of 3.8(±2.12%).

**Table 2:** Seroprevalence of paratuberculosis in six districts of western Uganda based on ELISA test results

District	Number tested (n)	Number positive	District apparent prevalence (%)	District true prevalence (%)	No. of herds tested	Positive herds	Herd Prevalence (%)	Within-herd Prevalence (%)
Mbarara	242	7	2.9	4.3	13	7	53.8	2.1
Ntoroko	180	9	5.0	8.5	9	7	77.8	7.3
Ntungamo	335	16	4.8	8	14	8	57.1	3.4
Kiruhura	402	8	2.0	2.5	15	5	33.3	1.6
Kabale	315	10	3.2	4.9	18	8	44.4	5.2
Bushenyi	341	8	2.3	3.3	24	5	20.8	3.2
**Total**	**1814**	**58**	**Av. 3.36**	**Av. 5.3**	**93**	**40**	**42.8**	**3.8**

**Fig. 1:**
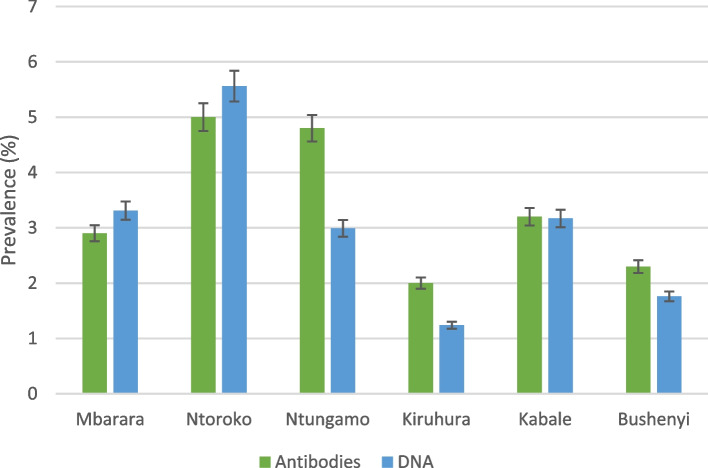
A graph to compare the prevalence estimates of MAP infection in the six districts using ELISA and RPA tests. The estimates by the two tests were almost at parity especially for samples from Kabale though ELISA resulted in higher estimates in three of the six districts

On the other hand, an apparent cow-level prevalence of 2.7% and a herd-level prevalence of 40.86% for herds which had at least one cow testing positive were obtained using RPA (Table 3). Similar to ELISA results, the prevalence based on MAP DNA detection was lowest in Kiruhura at 1.24% and highest in Ntoroko at 5.6% (Fig. 1), with a district level prevalence of 3(±1.5%). A true cow-level prevalence of 4.9% (95% CI: 3.5–6.7) and 3% (95% CI: 2.29–3.97) were obtained based on ELISA and RPA test methods respectively. The two tests were very close despite being based on different analytes. In three districts, ELISA showed higher prevalence estimates, while in two, RPA showed higher estimates but in one, the two were almost at parity (Fig. 1).

**Table 3:** Prevalence of *M. avium subspecies paratuberculosis* DNA in faeces of cattle in six districts of western Uganda

District	Number tested (n)	Number positive	District apparent prevalence (%)	District true prevalence (%)	No. of herds tested	Positive herds	Herd Prevalence (%)
Mbarara	242	8	3.31	3.7	13	7	53–85
Ntoroko	180	10	5.56	6.2	9	6	66.67
Ntungamo	335	10	2.99	3.3	14	6	42.86
Kiruhura	402	5	1.24	1.4	15	4	26.67
Kabale	315	10	3.17	3.6	18	9	50
Bushenyi	341	6	1.76	2	24	6	25
**Total (N)**	**1814**	**49**	**Av. 3.0**	**Av. 3.4**	**93**	**38**	44.17

For risk factor analysis, the intra-cluster (herd) correlation coefficient was used as a measure of contribution of herd variations to the overall variations in MAP infection. The estimated intra-cluster correlation coefficient by Fleiss-Cuzick kappa was 0.013 (95% CI: 0–0.125) meaning that the contribution of herd-based variations in MAP infection was only 1.3% of the total variations in the infection. This implied that ordinary logistic regression model other than mixed-effects model would suffice to predict the associations. Indeed, variance of random effects for the herd intercept was zero based on exploratory mixed-effects logistic model.

The analysis with the binary logistic regression model revealed only the length of the dry season exhibited significant association with MAP occurrence (*p* < 0.05) (Table 4).

**Table 4:** Binary logistic regression estimation of risk factors associated with the prevalence of paratuberculosis in six districts of Uganda

Dependent variable: MAP infection status					
Covariates	Odds ratio	Std. Err.	Z	***P*** -value	95% CI
**Breeding service**					
Bull used only on farm	1.000				
Bull is shared	1.577	0.703	1.02	0.306	0.659, 3.778
Artificial Insemination	2.797	2.232	1.29	0.198	0.659, 3.778
Artificial Insemination & Bull	1.383	0.622	0.72	0.471	0.573, 3.41
**Manure handling**					
Taken to plantation	1.000				
Used in plantation or sold	0.496	0.245	−1.420	0.156	0.188, 1.307
Left on the farm/pasture	1.027	0.269	0.100	0.918	0.615, 1.716
**New stock introduced**					
No	1.000				
Yes	1.172	0.307	0.600	0.546	0.700, 1.960
**Length of dry season**					
No or short dry spell	1.000				
Long dry spell	1.604	0.373	2.030	0.042	1.017, 2.531
**Tree shade density**					
Heavy canopy	1.000				
Intermediate	1.492	1.134	0.530	0.599	0.336, 6.619
Sparse	1.893	1.422	0.850	0.395	0.434, 8.252
**Water logging**					
No	1.000				
Yes	1.027	0.268	0.100	0.918	0.616, 1.714

The predicted probability of getting infected with MAP is 0.06 during the season of a long dry spell compared to 0.04 for a short or no dry spell. Other factors such as the breeding method, manure handling, or introduction of new stock did not exhibit significant association with paratuberculosis infection

## Discussion

This study aimed at determining the paratuberculosis infection status in selected districts in western Uganda and the associated risk factors.

The obtained results indicate that MAP is actively present in this region. These results do not differ significantly from those obtained in earlier studies in other parts of the country [21, 22]. This situation is also not different from other African countries such as Egypt which reported 12.6% (*n* = 500) MAP ELISA positive cattle [23], and Sudan which has recently reported an apparent seroprevalence of 6.3% (*n* = 175) in cattle in Khartoum State [24]. Countries without a national control program such as India have reported high individual animal seroprevalence rates of MAP ranging between 21.4–29.8% among the cattle population [25, 26]. The trend in India indicates an increase in MAP prevalence in domestic livestock from 11.4 to 44.2% from 1985 to March 2017 [27]. A similar trend can be envisaged in most African countries since no control strategies have been put in place to curb the spread of MAP infection. However, the scenario is different in countries that earlier had high prevalence but instituted control measures that brought the prevalence down such as Denmark and Japan [28–30]. Although paratuberculosis prevalence is still low in Ugandan cattle, there is a cause to worry as the prevalence at the herd level is very high. Based on ELISA, the herd-level prevalence was close to 45%, whereas, with the combined ELISA and RPA results, herd-level prevalence is 64%; this is an indication that there is high transmission between herds and the disease spread remains uncontrolled as there are presently no control or prevention measures in place which may exacerbate the situation. Currently, PTB is not among the diseases routinely examined at inspection points before the issuance of animal movement permits. This implies that the spread by animal movement could be one of the most important avenues for the spread of the disease among herds.

There were slight variations in the level of MAP detection between the serologic and molecular diagnostic tests. In some districts, the nucleic acid detection method identified more MAP-positive animals compared to the antibody method. However, overall, the ELISA had more positive results compared to the RPA test. These differences could be attributed to some animals that are shedding MAP in faeces but have not mounted a detectable immunological response due to the delay of the humoral immune response in paratuberculosis infection and others may have variations in shedding patterns or are intermittent shedders [31, 32]. From previous studies, the nucleic acid-based method detected more MAP-positive animals compared to the antibody method [33, 34] whereas, in another study, more positive animals were detected with serology as compared to the molecular method [35]. Generally, using a combination of both molecular and serologic test methods to diagnose for paratuberculosis improves ability to detect positive animals; and this was true for the current study.

The RPA diagnostic technique is fast and simple as it involves few pipetting steps. The test is minimally affected by the presence of inhibitors hence the extracted DNA can be used directly in the detection step with no further purification. All RPA reagents are stable at ambient temperature and the test involves few equipment requirements which can be adopted in a mobile laboratory setting at the point-of-care or in the field. However, during MAP DNA extraction, heating and bead beating is essential to ensure optimal yield of DNA from the faecal sample. It is also important to minimize debris during the extraction to avoid excess background. The extracted DNA need to be used immediately in the detection step to avoid possible degradation during storage. This is the first time the RPA test has been applied in a paratuberculosis study in Uganda since its validation [36] and the test has been effectively applied to screen animals for MAP. The portability of the suitcase lab as well as the speed of molecular test (40 min including the extraction protocol) have enabled the screening of large numbers of samples in a very short time and eliminate the storage problem.

Among the risk factors investigated, farms that experienced long dry spells were at higher odds of getting MAP infection compared to those that had shorter or no dry spells. The effect of prolonged drought could be attributed to nutritional stress as it is accompanied by lack of adequate feed and water, in addition to excessive heat. These could reduce the ability of animals to mount effective defence mechanisms against diseases, making them susceptible to MAP infection. Additionally, during dry seasons, animals from different herds and of different age groups congregate at water drinking points; this could facilitate disease transmission including paratuberculosis, which is mainly transmitted through the faecal-oral mode [37]. There were no significant differences in MAP infection among the different breeds of cattle in this study. On the other hand, factors such as manure handling, calf feeding, and housing methods were found to be associated with MAP infection in other studies [16]. However, we were unable to establish such association because the majority of the farms visited used extensive grazing practices, sometimes on rangeland, which minimizes the contact of animals with the infective faecal material from other animals as is the case in intensive management systems.

## Conclusion

This study has shown that paratuberculosis exists in all six districts of western Uganda, suggesting that the disease may be widespread across the region. The high herd-level prevalence and within-herd prevalence in some farms also indicate that MAP has been spreading between and within herds for many years. However, there is need to establish data on the prevalence of the disease in all parts of the country as this is necessary to lay strategies for MAP control and prevention. If no control measures are taken soon, paratuberculosis will continue to spread unchecked and is likely to become endemic in the country. Since MAP is suspected to be involved in the pathogenesis of Crohn’s disease and associated with many others in humans, there is need to carry out a human study to determine the level of human exposure to MAP and the occurrence of CD and Ulcerative Colitis in Uganda. The RPA as a diagnostic technique, can be adopted for wider use to improve disease detection and early reporting.

## Materials and methods

**Fig. 2:**
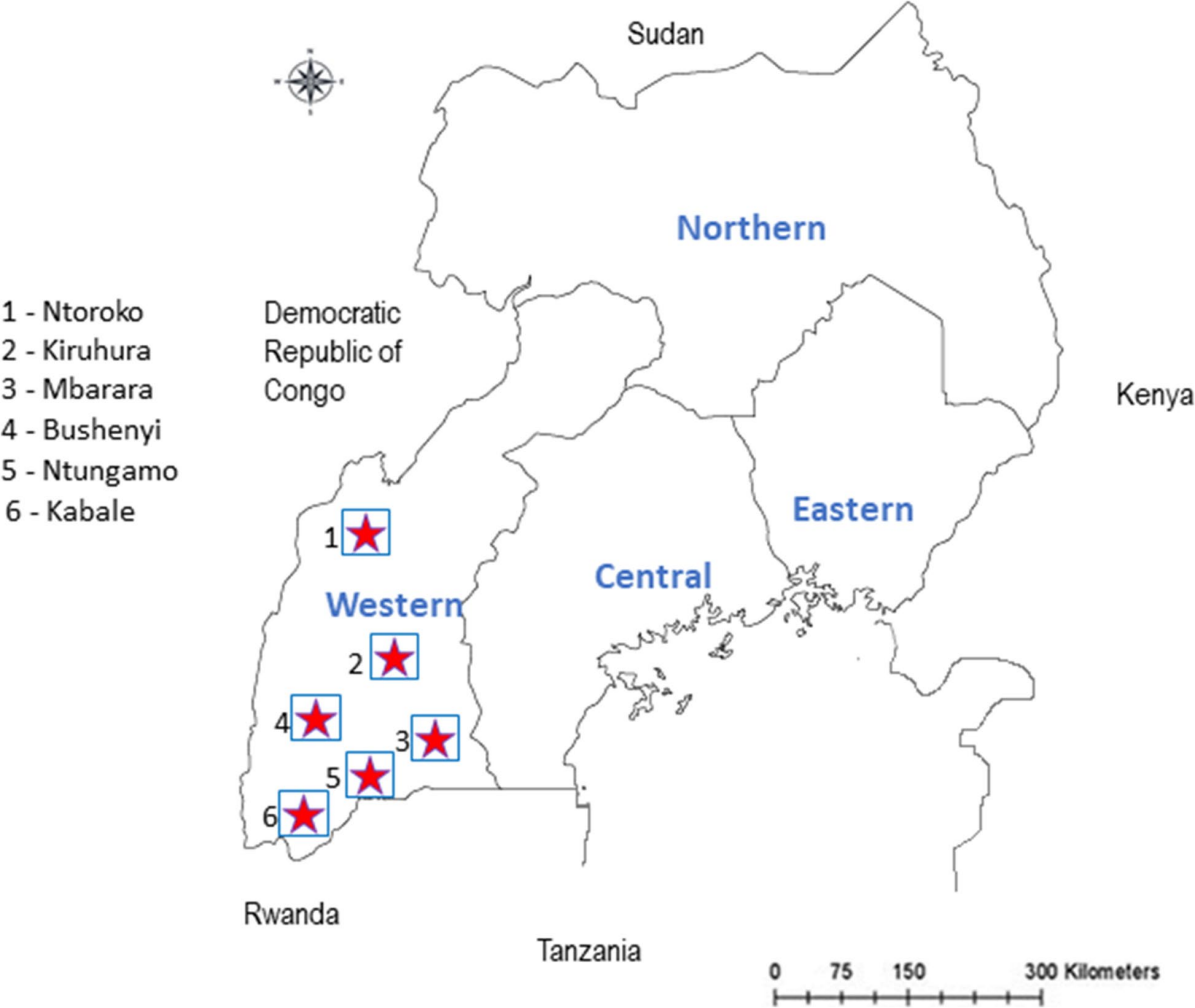
Map of Uganda showing districts sampled during the study. The location of the districts in which the study was carried out is shown by the red stars. (Source: Google maps)

To estimate the prevalence of MAP infection in cattle in western Uganda, a cross-sectional study covering six districts namely: Ntungamo, Kabale, Ntoroko, Mbarara, Kiruhura and Bushenyi (Fig. 2) was carried out between October, 2019 and March 2021. These districts lie within the south and western highland agro-ecological zones. They experience a bimodal rainfall pattern and the vegetation mostly includes tropical woodland, bushland as well as savannah grassland [38]. Farmers in this region mainly practice extensive grazing of cattle with fences to demarcate farming units and majorly graze on natural pastures with limited intensive production.

A multistage cluster sampling technique was employed and the sample size was estimated using the WINPEPI programme (Version 3.18) assuming an expected prevalence level of 8% [21], design effect of two and an acceptable difference of 4% [39] at 95% level of confidence. The number of animals sampled from each district was calculated depending on the population size and the average size of each cluster/herd. The total number of samples taken from each district, n, was the sum of all the samples taken from each of the clusters. Within each cluster, all cattle of two years and above were sampled. Thus, the total number (N) of samples in the study was calculated as follows: N = n1 + n2 + n3 + n4 + n5 + n6, representing all the six districts.

To investigate the effects of various risk factors, questionnaires were used to collect information from the cattle owners or their representatives about history of Johne’s disease-related symptoms, breeding practices, calf feeding practices, grazing practices, manure handling as well as environmental factors such as tree shade canopy on pastures, length of the dry season and water logging; that may contribute to MAP introduction, favour environmental persistence and spread in animals (See Additional File 1).

Blood and faecal samples were collected from all cattle after proper restraint following the recommended handling procedures. Approximately 4 ml of blood were collected from the jugular or coccygeal vein using a sterile vacutainer needle and a plain vacutainer tube (BD vacutainer®, Franklin Lakes, NJ USA), labelled and stored in a mobile fridge (Dometic, Model CDF 26, Hong Kong, China) at 2–8 °C. Faeces was collected from the same animal directly from the rectum using clean arm-length or powder-free latex gloves, put in a 50 ml sample collection bottle (Sarstedt, Nümbrecht, Germany), labelled and stored in ice-cooled boxes for transportation to the laboratory. At the laboratory, serum was harvested into 1.5 ml cryovials, and stored at − 20 °C for subsequent testing with ELISA while faeces was also stored at − 20 °C for subsequent MAP DNA extraction and detection using Recombinase Polymerase Amplification (RPA).

### Detection of *Mycobacterium avium* subsp. *paratuberculosis* antibodies

To test for the presence of MAP antibodies in sera, an Enzyme-Linked Immunosorbent Assay (ELISA) was performed using a commercial test kit (IDEXX® Paratuberculosis Screening, IDEXX Laboratories, Inc., Westbrook, USA), according to the manufacturer’s instructions. Briefly, the serum and all test reagents were allowed to thaw at room temperature (RT) for about 30 min. The samples and controls sera (two positive and one negative) were diluted at 1:20 using dilution solution in the 96-well uncoated plates in the presence of *Mycobacterium phlei* and mixed by agitating on a shaker and incubated for 15 min at RT. One hundred microlitres of the pre-incubated samples and controls were dispensed into MAP antigen-coated wells in a 96-well microtiter plate, mixed gently by rocking for 5 seconds then covered with aluminium foil and incubated at RT for 45 min. The plate was then emptied by pouring off the contents in a fling and tapped on a blotting paper to remove excess fluid. The plate was washed thrice with approximately 300 μl of diluted wash buffer (1:20) for each well and blotted again. One hundred microliters of diluted conjugate (1:100) were added into each well, homogenised, covered and incubated at RT for 30 min. A second washing step followed and 100 μl of Tetramethyl Benzidine (TMB) solution were added to each well. The plate was covered with foil and incubated in the dark for 10 min. To stop the reaction, 100 μl of stop solution were added in each well. The optical density (OD) was read using a spectrophotometer reader (SPECTROstar Nano, S/N 601–1258 - BMG LABTECH, Ortenberg, Germany) at a spectrum absorbance of 450 nm. The OD of the samples and controls were entered into Microsoft Excel spreadsheets for analysis. The sample to positive ratios (S/P) were calculated from the mean OD of the positive control (x̄PC) and the OD of the negative control (xNC) as; S/P = (OD of sample – xNC) / (x̄PC – xNC) *100.

The manufacturer set the S/P cut-off point for a sample to be considered positive at ≥55%.

### Detection of MAP DNA from faeces

#### DNA extraction

MAP DNA was extracted from faecal samples using the SwiftX kit (Xpedite, Munich, Germany) in a mobile suitcase laboratory setting according to Hansen et al. [40]. Briefly, 100 mg of faeces were added to 500 μl of lysis buffer in a Precelly’s SK 38 (Bertin Corp., Rockville, MD, USA) tube including a negative extraction control, and vortexed for 1 min. Sixty microlitres of magnetic beads were added to the sample and mixed well for 10 s by vortexing or inverting. The tubes were then incubated in a heat block at 95 °C for 15 min; removed from the heat block every 2 min and vortexed. The tubes were removed from the heat block, either shaken down or tapped on a bench to remove condensate from the lid, transferred to a magnetic stand with loosened caps, and incubated at RT for 2 min. Then the sample tubes were opened carefully and 10 μl of clear solution were transferred to a 1.5 ml tube containing 40 μl of water (molecular biology grade) to obtain a dilution of 1:5. The diluted sample containing the extracted DNA was directly used in the following step.

### Recombinase polymerase amplification (RPA)

To test for presence of MAP in faeces, a Recombinase Polymerase Amplification (RPA) test protocol was performed as previously described [36]. In brief, the dried oligos mix (primer and probe) and positive control (molecular standard) were reconstituted by addition of 520 μl and 300 μl of molecular biology grade water respectively, and the protocol was performed as follows: to RPA exo kit, 29.5 μl of rehydration buffer, 13 μl of oligos mix and 2.5 μl of magnesium acetate (280 mM) were dispensed into the lid of each of the tubes, then 5 μl of the extracted DNA were added to the mix in the lid before closing the tubes. Negative and positive controls were included in each test run. The tubes were then centrifuged, vortexed and again centrifuged in this order and placed into a T8 – ISO tube scanner (Axxin, Fairfield, Australia). The T8 machine was pre-set to start automatically once the lid was closed. The test could be monitored on the screen as it progresses in a graphical form. After 4 min, the lid opened automatically; the strip was removed shortly, vortexed, centrifuged and placed back into the machine in the same order. The machine detects fluorescence resulting from the degrading probe. The detection process is completed after 15 min and the machine indicates ‘test completion’. Test results were viewed directly on the screen and downloaded into MS spreadsheets for subsequent analysis.

### Data analysis

ELISA and RPA results were analysed using Microsoft spreadsheets (MS Excel). The true prevalence for the ELISA and RPA tests was calculated using EPITOOLS programme [41, 42] at 95% confidence level taking into account the sensitivities and specificities of the two tests and the respective confidence intervals (CI). The ELISA kit (IDEXX Paratuberculosis screening, IDEXX Laboratories, Inc., Westbrook, USA), had an estimated sensitivity (Se) of 51.4% and specificity (Sp) of 99.3% [43]; whereas the Se and Sp of the RPA test were taken as 89.5 and 100% respectively as reported by Hansen and co-workers [36]. Data for risk factor analysis was also entered into MS Excel and statistically analysed for association with occurrence of MAP on the farm using the Stata 15.1 statistical programme (Texas, USA).

## Supplementary Information

**Additional file 1. **Epidemiological tool for the survey of *Mycobacterium avium* subsp. *paratuberculosis* (MAP) infection and associated risk factors among cattle herds in Uganda. Description: Survey tool used to generate epidemiological data from surveyed farms for analysis of risk factors associated with paratuberculosis infection

**Additional file 2.** MAP Survey Characteristics. Description: MAP infection status of cattle based on serological and molecular tests and the corresponding epidemiological information in the six surveyed districts of western Uganda

## Data Availability

All data are available in the manuscript text and in the supplementary information files.

## Abbreviations

min: minute(s)
s: seconds
ml: millilitres(s)
^0^C: degrees Celsius
μl: microlitre(s)
nm: nanometres
mg: milligram(s)
Fig.: Figure
Av.: Average
Sp: Specificity
Se: Sensitivity
