# Supplementary material for: Molecular and serological survey of paratuberculosis in cattle in selected districts of Western Uganda
Source: BMC Vet Res. 2022 Dec 14;18:438. doi: 10.1186/s12917-022-03535-7 (PMC9749330; doi:10.1186/s12917-022-03535-7)
Supplement: Supplementary file 1 — Additional file 1. Epidemiological tool for the survey of Mycobacterium avium subsp. paratuberculosis (MAP) infection and associated risk factors among cattle herds in Uganda. Description: Survey tool used to generate epidemiological data from surveyed farms for analysis of risk factors associated with paratuberculosis infection [file 12917_2022_3535_MOESM1_ESM.docx]

**Epidemiological tool for the survey of *Mycobacterium avium* subsp. *paratuberculosis* (MAP) infection and associated risk factors among cattle herds in Uganda**

This questionnaire is intended for the collection of information that will promote our understanding of the role of different factors that promote the spread of Paratuberculosis or Johne’s disease. It is not meant for the collection of information for tax assessments or any other political reason. As a respondent, you are free to participate or decline participation without any risk of punishment of whatever sort or loss of privileges. Due to the benefits that research contributes to society, we request your voluntary participation to respond to these questionnaires.

Please respond to these questions and ask if it is not clear to you

1. Farmer’s information

Name of the farmer: ……………………………………………………. Village: …………………………………….

Parish: ……………………………………………………… Sub county: …………………………………………………

District: ……………………………………………………

1. Animal characteristics
2. How many cattle do you have? Total number: ………………… Female: ……………………

Males (bulls and steers): ……………………………..

1. Age groups of the cattle kept; (a) Below 6 months…………. (b) 6-12 months………….

(c) 1-2 years ……………… (d) 2-3 years ……………… (e) Above 3 years ………………………

1. Herd characteristics
2. What cattle breeds do you keep on the farm? ……………………………………………………
3. For how long have you kept these breeds of cattle in the farm? ………………………..
4. Has any of the animals suffered from prolonged diarrhea with body wasting?

Yes: …………. No: ……………….

1. If yes, how old were the affected cattle? …………………… the breed: …………………….
2. Were you able to treat and cure the affected cattle? …………………………………………...
3. Johne’s disease prevalence
4. How many heads of cattle do you have? ……………………………………………………………..
5. Number of cattle sampled from the herd: ……………………………………………………………
6. The ecology of *Mycobacterium avium* subsp. *paratuberculosis*
7. Rainfall or drought pattern in the area (Refer to meteorological data)

- Do you experience long dry spells in your area? Y/N …………….
- How long is the long rainy season? …………….. the long dry season ………………….

1. Vegetation cover of the farm?

- Do you have tree shades on your farm? Y/N ………….
- How do you rate tree shades on your farm? a) Sparse: …………………….

1. Heavy canopy: ……………… c) Intermediate: ……………………….

- Do you graze on natural or planted pasture? …………………………

1. Drainage of the farm

- Do you have waterlogging on the farm? (Is the farm well drained?) ……

…………………………………………………………………………………………………………….

1. Water source for the cattle

What is the source of water for the animals on the farm? (tick available water source)

1. Borehole
2. Deep well
3. Valley dam (a) Individually owned ……………. (b) Communally owned ……………….
4. Stream(s)
5. River
6. Grazing practices (tick practice noted)

i. Paddocking ii. Communal grazing iii. Rangeland grazing iv. Transhumance

v. Grazing in rented area vi. Periodic bush burning

1. Stocking density estimation in paddocked farms
2. Number of cattle kept on the farm
3. Farm size in hectares
4. Stocking density
5. Stocking and restocking of the farms
6. Have you introduced new stock on the farm? Y/N
7. When was/were cattle introduced? In the last: 6 months ………….. One year …………

Two years ……………………. Others (specify) ……………………………

1. Breeding practices: Where do you get bulls for breeding?
2. Privately owned bull and is only used in the farm ……………………………………………….
3. Shared bull from other farmers …………………………………………………………………………..
4. Artificial insemination ……………………………………………………………..
5. Both artificial insemination and bull ……………………………………..
6. Calf feeding practices: How do you feed calves on the farm?
7. Calves suckle and walk with their dam during grazing
8. Calves suckle and remain at the homestead
9. Calves are bucket fed
10. Culling practices: How do you cull animals from your herd of cattle?
11. Slaughter them
12. They are sold alive to any potential buyers
13. Common beliefs regarding poor animal health such as loss of body condition (unthriftiness)
14. What diseases associated with prolonged diarrhea and weight loss do you know?

……………………………………………………………………………

…………………………………………………………………………..

1. Do you know the causes of these diseases you have mentioned?

……………………………………………………………………………………………..

1. What traditional means of treatment do you have for the diseases mentioned?

………………………………………………………………………………………

1. Any other traditional knowledge that explains the cause of such a disease and how traditional knowledge can be applied to treat these diseases
2. Other than cattle, which other livestock do you keep on the farm?

……………………………………. …………………………..…………… ……………………………………………

1. Have any of these livestock also suffered from chronic wasting disease with loose stool that failed to respond to treatment? Y/N ……………………
2. If Yes, which stock and how many? …………………………… ………………………………
3. Did you ever find any solution to the problem? Y/N …………………………..
4. Have you ever heard of Johne’s disease or Paratuberculosis? Y/N …………………..
5. If Yes, from whom? ……………………………………
6. Are there wildlife within or around your premises? Y/N …………………………..
7. If Yes, which species of wildlife? …………………………….. ……………………………………….
8. How do wildlife affect farming practices on your farm?

……………………………………………………………… ……………………………………………………………

1. How do long dry seasons affect your husbandry practices?

………………………………………………………………………………………………………………………………….

1. How do long rain seasons affect your farming practices?

…………………………………………………………………………………………………………………………………

1. How do you handle accumulated manure on your farm?
2. Left in the farm/pasture
3. Taken to plantations
4. Used in plantations or sold

**Thank you for your responses**
